# Supplementary material for: SFMBT2-Mediated Infiltration of Preadipocytes and TAMs in Prostate Cancer
Source: Cancers (Basel). 2020 Sep 22;12(9):2718. doi: 10.3390/cancers12092718 (PMC7565541; doi:10.3390/cancers12092718)
Supplement: Supplementary file 1 [file cancers-12-02718-s001.zip › Supplementary (1).docx]

**Supplementary Figures**

**Figure S1. Chemokine-mediated migration of preadipocytes and TAMs.** (A) Culture media (CM) from LNCaP cells stably transfected with SFMBT2 shRNA promote migration of 3T3-L1 preadipocytes. However, addition of antibodies against CXCL8, CCL2, CXCL10, and CCL20 and a mixture of antibodies reduces migration of 3T3-L1 preadipocytes. After incubation of 3T3-L1 preadipocytes with antibodies against indicated chemokines in culture media from LNCaP cells stably transfected with SFMBT2 (shSFMBT2) or control shRNA (shCont), transwell assay was performed. Migrating 3T3-L1 preadipocytes were stained with neutral red. Representative images are shown. (B) Treatment of CXCL8, CCL2, CXCL10, and CCL20 promotes migration of 3T3-L1 preadipocytes. Addition of antibodies against CXCL8, CCL2, CXCL10, and CCL20 reduces migration of 3T3-L1 preadipocytes. After incubation of 3T3-L1 preadipocytes with antibodies against indicated chemokines in RPMI 1640 media, transwell assay was performed. Migrating 3T3-L1 preadipocytes stained with neutral red. Representative images are shown. (C) Culture media from LNCaP cells stably transfected with SFMBT2 shRNA promote migration of TAMs polarized from Raw264.7 cells. However, addition of antibodies against CXCL8, CCL2, CXCL10, and CCL20 and a mixture of antibodies reduces migration of TAMs. After incubation of TAMs with antibodies against indicated chemokines in culture media from LNCaP cells stably transfected with SFMBT2 or control shRNA, transwell assay was performed. Migrating TAMs were stained with neutral red. Representative images are shown. (D) Treatment of CXCL8, CCL2, CXCL10, and CCL20 promotes migration of TAMs polarized from Raw264.7 cells. However, addition of antibodies against CXCL8, CCL2, CXCL10, and CCL20 reduces migration of TAMs. After incubation of TAMs with antibodies against indicated chemokines in RPMI 1640 media, transwell assay was performed. Migrating TAMs were stained with neutral red. Representative images are shown.

**Figure S2. SFMBT2 regulates** **migration of preadipocytes and TAMs in PC3 cells.** (A, B) Culture media (CM) from PC3 cells promote migration of 3T3-L1 preadipocytes and TAMs polarized from Raw264.7 cells. After incubation of 3T3-L1 preadipocytes or TAMs in culture media from LNCaP cells stably transfected with control RNA (shCont), SFMBT2 shRNA (shSFMBT2), and PC3 cells, transwell assay was performed. Migrating 3T3-L1 preadipocytes and TAMs were stained with neutral red and counted. (C) Overexpression of SFMBT2 tagged with HA suppresses up-regulation of CXCL8, CCL2, CXCL10, and CCL20 expression in PC3 cells endogenously expressing a low level of SFMBT2. Western blotting was performed using anti-CXCL8, anti-CCL2, anti-CXCL10, anti-CCL20, and anti-HA antibodies. Anti-tubulin antibody was used for loading control. Western blots were analyzed quantitatively using ImageJ program. (D) Culture media from PC3 cells overexpressing SFMBT2 suppress migration of 3T3-L1 preadipocytes and TAMs polarized from Raw264.7 cells. Transwell assay was performed. Migrated cells were stained with neutral red and counted. Representative images are shown. All data represent mean ± S.E.M. Significance values were * P≤0.05, ** P≤0.01, and *** P≤0.005.

**Figure S3.** **Expression level of SFMBT2, Pref-1, CD24, CD29, CD163, CD206, C/EBPα, FABP4, and Sca-1 in tumor xenograft.** LNCaP cells stably transfected with control (shCont) or SFMBT2 shRNA (shSFMBT2) were implanted subcutaneously into the flank of nude mouse. After 4 weeks, stably RFP-expressing 3T3-L1 cells were injected into the tail vein of the same mouse. At 1 week later, tumor was harvested and RNA was analyzed. Expression of SFMBT2 is decreased, but marker gene expressions of preadipocytes (Pref-1, CD24, CD29, Sca-1) are increased in tumor. However, marker gene expressions of mature adipocytes are not changed (C/EBPα) or slightly increased (FABP4). In addition, marker gene expressions of TAMs (CD163, CD206) are increased in tumor. All data represent mean ± S.E.M. Significance values were * P≤0.05, ** P≤0.01, and *** P≤0.005.

**Figure S4.** **No enrichment of SFMBT2 to CXCL8, CCL2, CXCL10, and CCL20 gene promoters in LNCaP cells.** ChIP assay was performed using anti-SFMBT2 antibody. The occupancy of each protein was determined with quantitative PCR in gene promoters encompassing potential YY1 binding sites. ChIP was performed using normal IgG as a control.

**Figure S5. Preadipocytes and TAMs promote migration and invasion of prostate cancer cells through up-regulation of IL-6.** (A, C) Culture media (CM) from 3T3-L1 preadipocytes or TAMs promote migration of LNCaP cells stably transfected with SFMBT2 shRNA. However, addition of anti-IL-6 antibody suppresses migration of LNCaP cells stably transfected with SFMBT2 shRNA. After incubation of LNCaP cells stably transfected with SFMBT2 (shSFMBT2) or control shRNA (shCont) with anti-IL-6 antibody in culture media from 3T3-L1 preadipocytes or TAMs, transwell assay was performed. Migrating LNCaP cells were stained with neutral red. Representative images are shown. (B, D) Culture media (CM) from 3T3-L1 preadipocytes or TAM promote invasion of LNCaP cells stably transfected with SFMBT2 shRNA. However, addition of anti-IL-6 antibody suppresses invasion of LNCaP cells stably transfected with SFMBT2 shRNA. After incubation of LNCaP cells stably transfected with SFMBT2 or control shRNA with anti-IL-6 antibody in culture media from 3T3-L1 preadipocytes or TAMs, transwell assay was performed. Invading LNCaP cells were stained with neutral red. Representative images are shown.

**Figure S6. Knockdown of SFMBT2 results in up-regulation of TNFα expression. (A)** Expression of TNFα was analyzed by quantitative RT-PCR in LNCaP cells stably transfected with control (shCont) or SFMBT2 shRNA (shSFMBT2). (B) Secreted level of TNFα was analyzed by ELISA in LNCaP cells stably transfected with control or SFMBT2 shRNA. (C) Overexpression of SFMBT2 suppresses TNFα gene expression in LNCaP cells. Gene promoter activity was analyzed in LNCaP cells transfected with luciferase reporter containing TNFα gene promoter. (D) Enrichment of SFMBT2 to TNFα gene promoter in LNCaP cells. ChIP assay was performed using anti-SFMBT2 antibody. The occupancy of each protein was determined with quantitative PCR in gene promoters encompassing potential YY1 binding sites. ChIP assay was performed using normal IgG as a control. (E) NF-κB activation is required for up-regulation of TNFα gene expression in LNCaP cells stably transfected with SFMBT2 shRNA. Expression of TNFα was analyzed by quantitative PCR in LNCaP cells stably transfected with control or SFMBT2 shRNA with or without BAY 11-7085 treatment. All data represent mean ± S.E.M. Significance values were ** P≤0.01 and *** P≤0.005.

**Figure S7. Uncropped western blot figures of Figure 5D (A) and Figure S2C (B).**

**Supplementary Table**

**Table S1. Oligonucleotides used in this study.**

**Table S2. Antibodies used in this study.**

**Table S3. Chemokines and cytokines used in this study.**

**Table S4. Characteristics of prostate cancer patients in tissue array used in this study.**

**Table S5. Relationship between expression level of SFMBT2 and Gleason scores.** Intensity values of images from whole tissue were analyzed with ImageJ software (NIH). In accordance with a previous report [[26](https://www.ncbi.nlm.nih.gov/pmc/articles/PMC5217015/#R72)], the images were scored based on signal intensity (no staining = 0, weak staining = 1, moderate staining = 2, strong staining = 3) and the extent of stained cells (0% = 0, 1~10% = 1, 11~50% = 2, 51~80% = 3, 81~100% = 4). Scoring was done by two reviewers, who were blind to the results. The final score was determined by multiplying the intensity scores with the extent of scores of stained cells. The final scores are in a range from 0 to 12. If the score is 0 to 4, it is called “SFMBT2 low”. If the score is 5 to 8, it is called “SFMBT2 moderate”. A score of 8~12 is called “SFMBT2 high”. A Kruskal-Wallis test was used to calculate P values.
