# Supplementary material for: SFMBT2-Mediated Infiltration of Preadipocytes and TAMs in Prostate Cancer
Source: Cancers (Basel). 2020 Sep 22;12(9):2718. doi: 10.3390/cancers12092718 (PMC7565541; doi:10.3390/cancers12092718)
Supplement: Supplementary file 1 [file cancers-12-02718-s001.zip › supplement 0818.pdf]

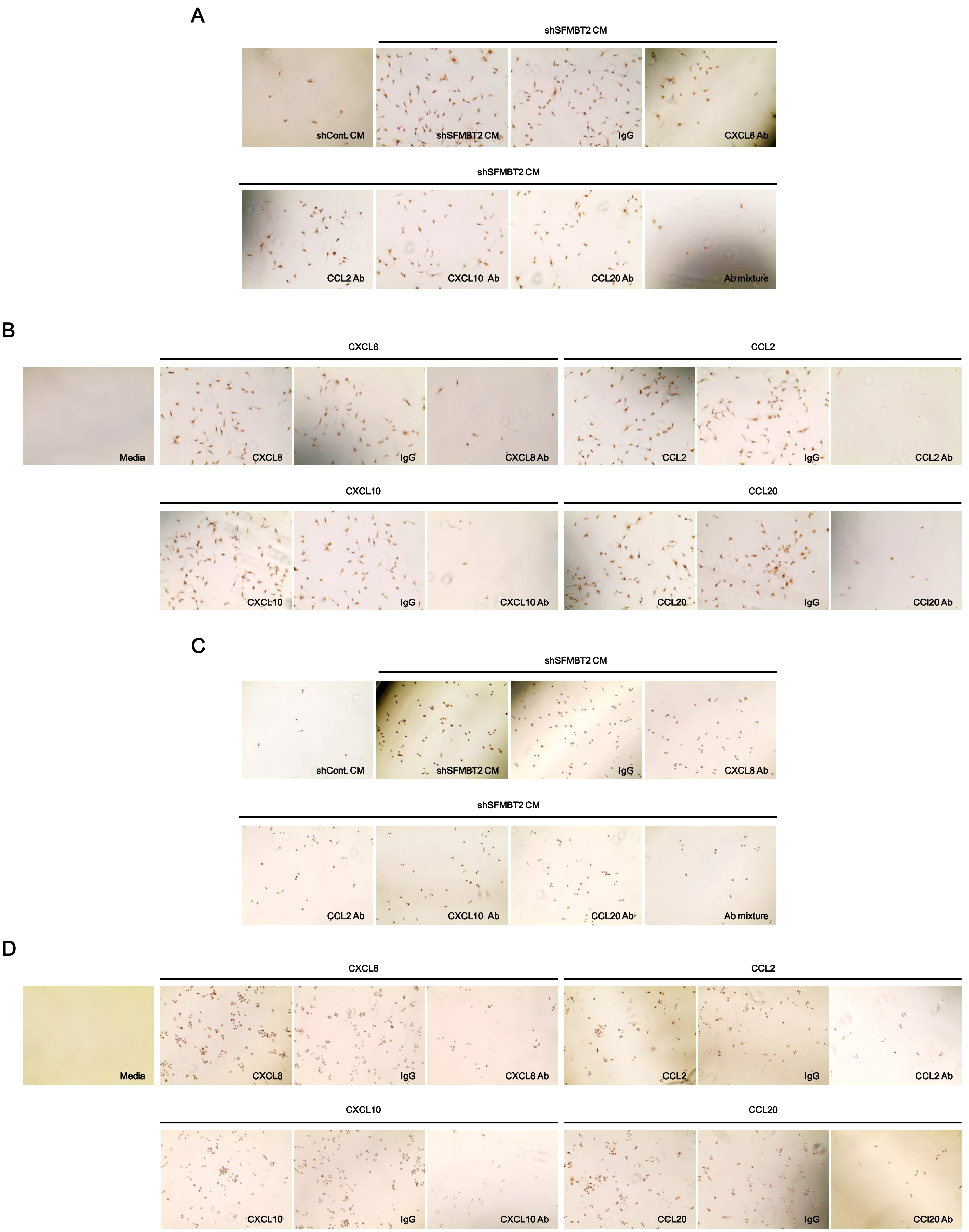

Supplementary Figure S1.

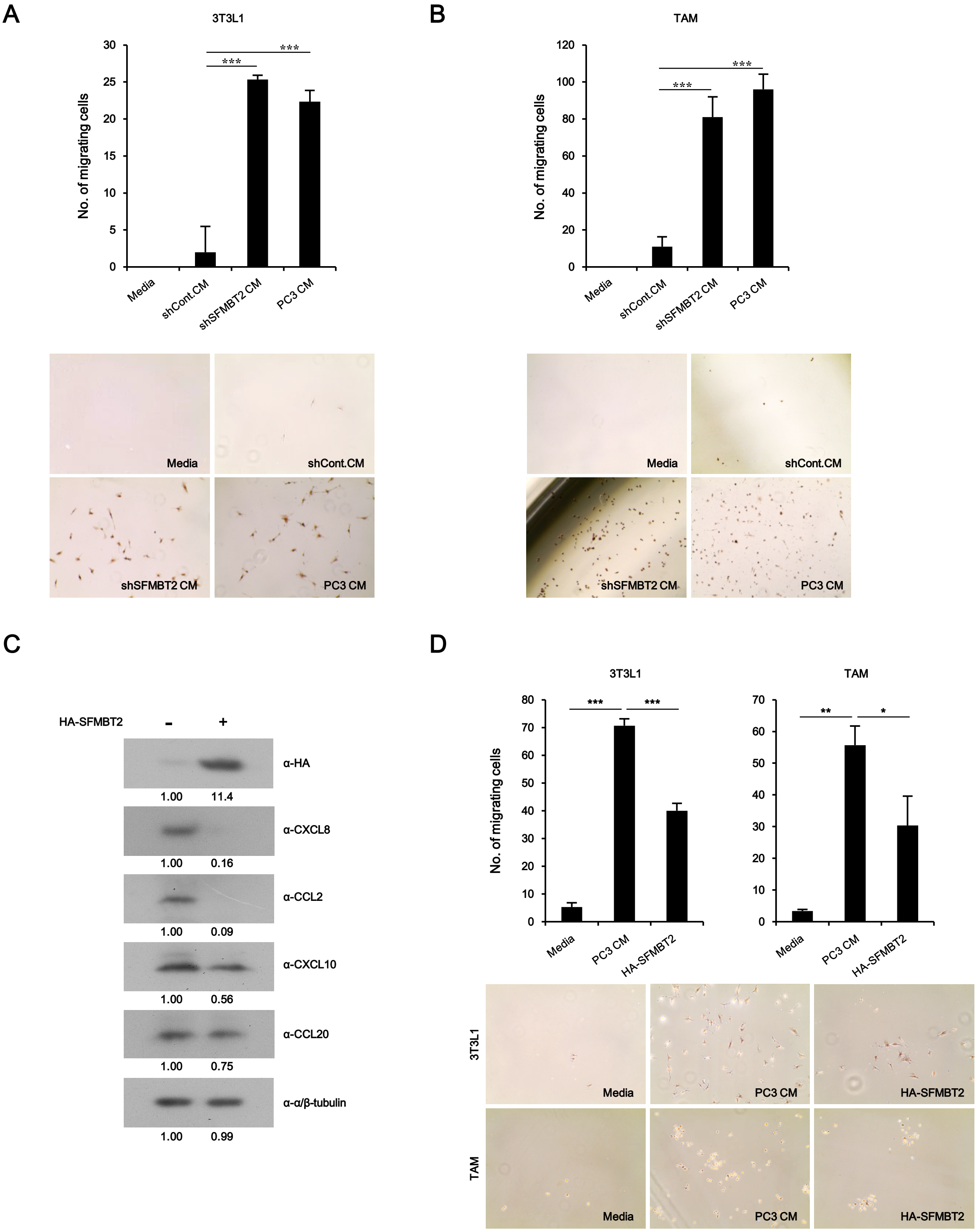

Supplementary Figure S2.

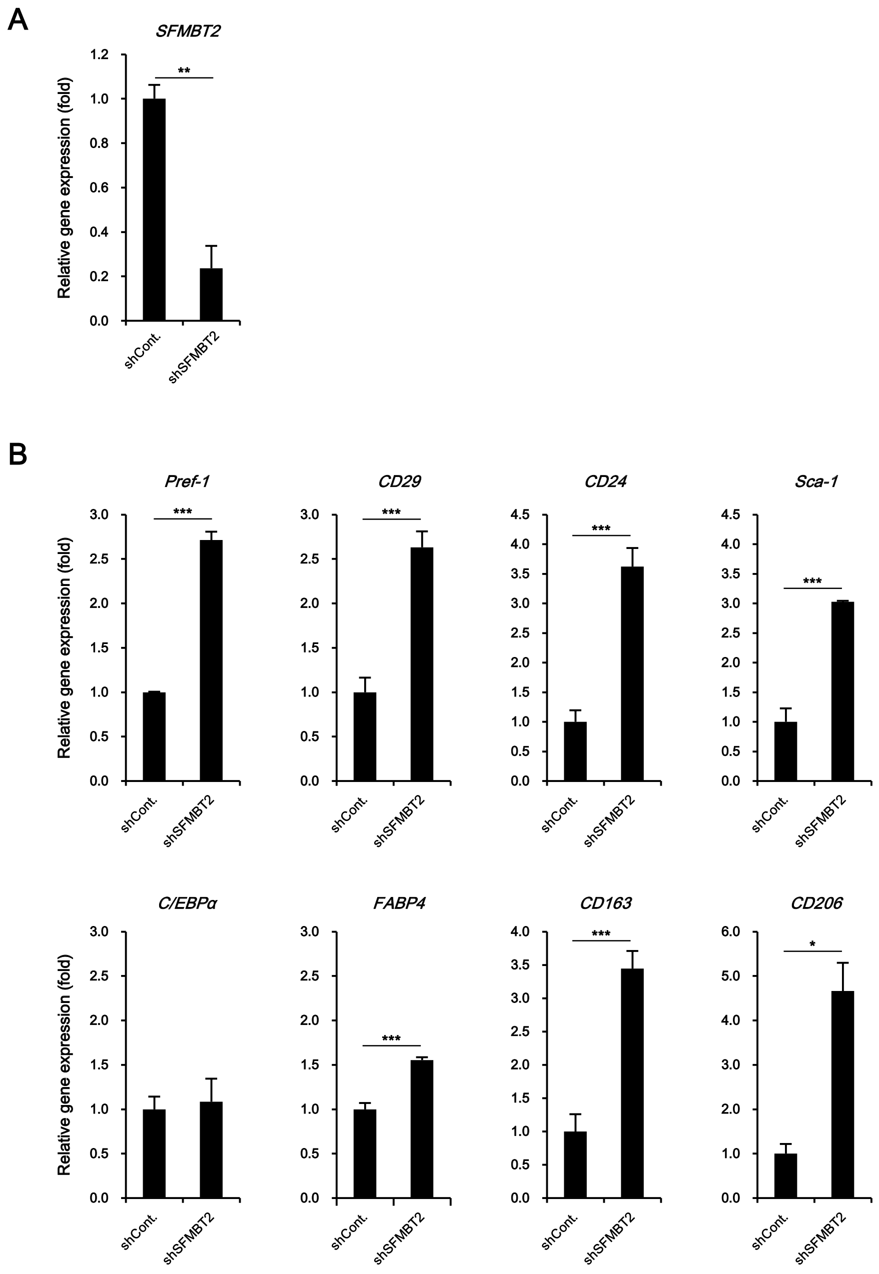

Supplementary Figure S3.

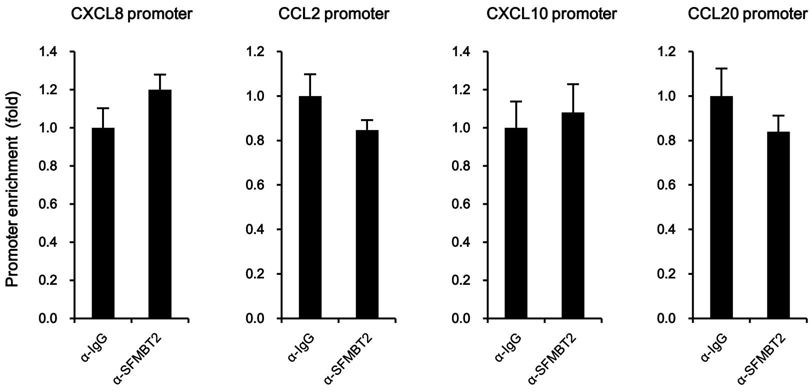

Supplementary Figure S4.

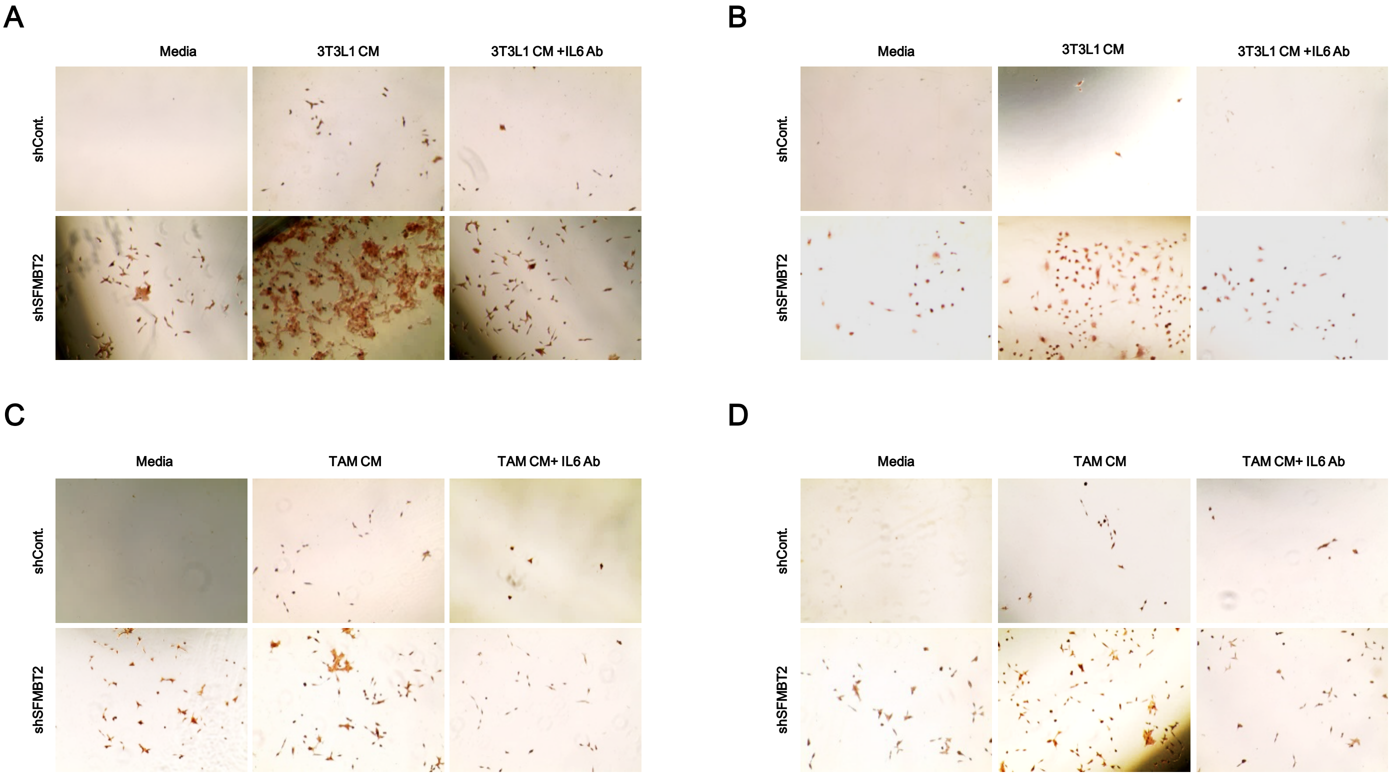

Supplementary Figure S5.

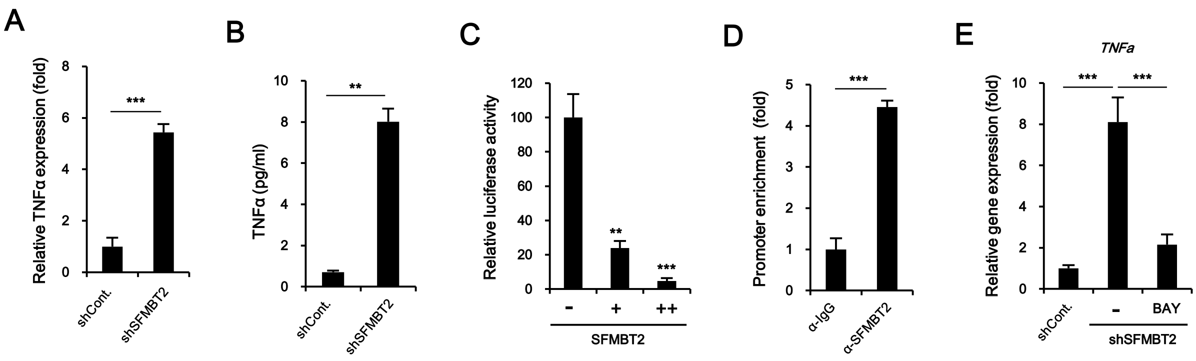

Supplementary Figure S6.

Full blots from Figure 5D (A) and Figure S2C (B)

A

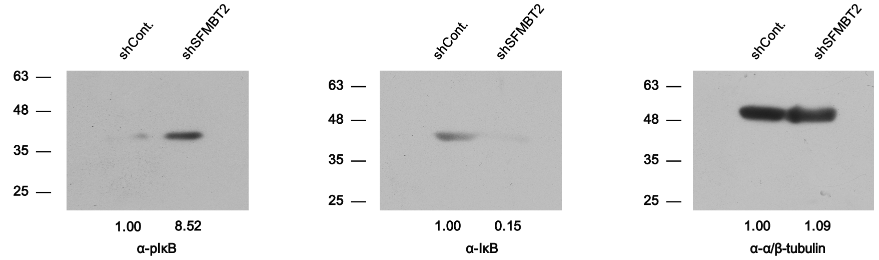

B

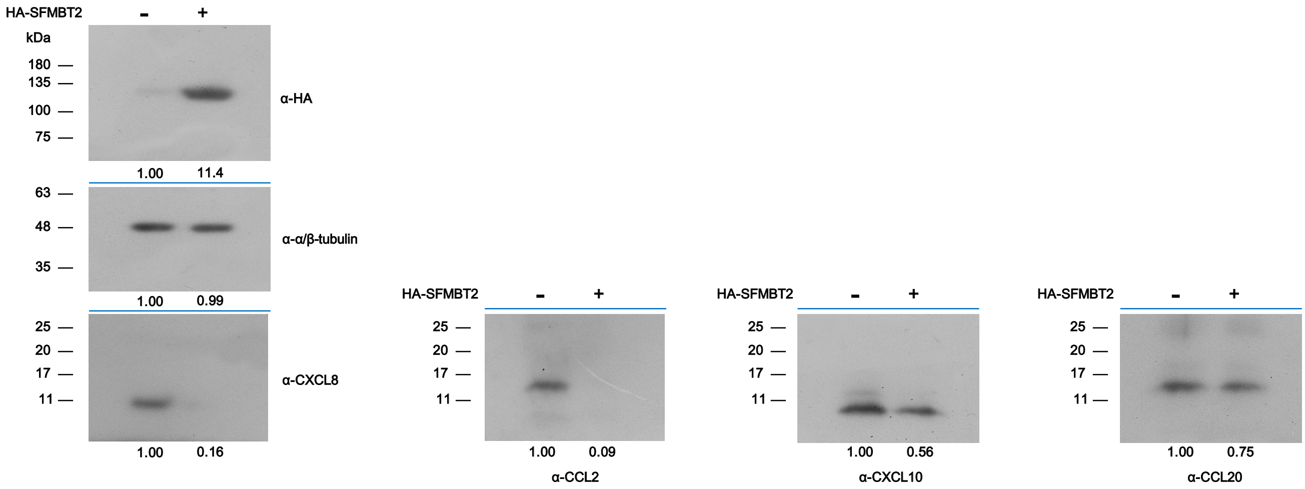

Supplementary Figure S7.

Table S1. Oligonucleotides used in this study.

Gene	Forward Primer	Reverse Primer
**qRT-PCR Primers**		
hSFMBT2	5’- TGACGTAGTCATCGCGGATTT -3’	5’- ACCAGTCAAGTCACGTATGAGAA -3’
hCXCL8	5’- ACTGAGAGTGATTGAGAGTGGAC -3’	5’- AACCCTCTGCACCCAGTTTTC -3’
hCCL2	5’- ATCACCAGCAGCAAGTGTC -3’	5’- AGGTGGTCCATGGAATCCTG -3’
hCXCL10	5’- CAGCAGAGGAACCTCCAGTC -3’	5’- GCAGGTACAGCGTACAGTTC -3’
hCCL20	5’- TGCTGTACCAAGAGTTTGCTC -3’	5’- CGCACACAGACAACTTTTTCTTT -3’
hTNFα	5’- GAGGCCAAGCCCTGGTATG-3’	5’- CGGGCCGATTGATCTCAGC -3’
h18s rRNA	5’- GTAACCCGTTGAACCCCATT -3’	5’- CCATCCAATCGGTAGTAGCG -3’
mIL6	5’- TAGTCCTTCCTACCCCAATTTCC-3’	5’- TTGGTCCTTAGCCACTCCTTC-3’
mCCR2	5’- ACACCCTGTTTCGCTGTAGG -3’	5’- GATTCCTGGAAGGTGGTCAA -3’
mCCR4	5’- GGAAGGTATCAAGGCATTTGGG -3’	5’- GTACACGTCCGTCATGGACTT -3’
mCCR6	5’- CTGCACTAGTGAGCGTGTGG -3’	5’- ACCTCTTCTAGGGAGCATGG -3’
mCXCR1	5’- ATTCGTGTGCGTGGGCATCT-3’	5’- CCCGCAAGGTCATCCGAAAA -3’
mCXCR2	5’- GACACAGCTCCAGTTAGGGATG -3’	5’- CGAGGTGCTAGGATTTGAGC -3’
mCXCR3	5’- GCTGTAGCCGATGTTCTGCTGGTG -3’	5’- TGCACTATGCTCAGATATCTGTC -3’
mPref-1	5’- AGTGCGAAACCTGGGTGTC -3’	5’- GCCTCCTTGTTGAAAGTGGTCA -3’
mCD24	5’- TTCTGGCACTGCTCCTACC -3’	5’- GCGTTACTTGGATTTGGGGAA -3’
mCD29	5’- ACTGTGATGCCGTATATTAGCAC -3’	5’- GATATGCGTTGCTGACCAACA -3’
mCD163	5’- AAGCATTACTGTCATCATAG -3’	5’- CTCCACCTACAAGTCTAA -3’
mCD206	5’- CAGGTGTGGGCTCAGGTAGT -3’	5’- TGTGGTGAGCTGAAAGGTGA -3’
mSca-1	5’- ACAGCACAGGCAGGAAGACT -3’	5’- TCGGTATTATCTTCGGGGC -3’
mC/EBPα	5’- TGGACAAGAACAGCAACGAG -3’	5’- TCACTGGTCAACTCCAGCA -3’
mFABP4	5’- AAGGTGAAGAGCATCATAACCCT -3’	5’- TCACGCCTTTCATAACACATTCC -3’
m18s rRNA	5’- AGTCCCTGCCCTTTGTACACA -3’	5’- CGATCCGAGGGCCTCACTA -3’
**ChIP Primers**		
CXCL8 (p65)	5’- GGGCCATCAGTTGCAAATC -3’	5’- TTCCTTCCGGTGGTTTCTTC -3’
CCL2 (p65)	5’- TGGAAAAAGTGTCTCGTCCTGAC -3’	5’- GCTGCTGTCTCTGCCTCTTATTGA -3’
CXCL10 (p65)	5’- AGGAGCAGAGGGAAATTCCGTAAC -3’	5’- AACGTGGGGCTAGTGTGCCA -3’
CCL20 (p65)	5’- TGAGGAAAAAGCAGGAAGTTT -3’	5’- GTACACAGAAGGCGTGTTGC -3’
CXCL8 (YY1)	5’- CCCCCACATTACTCAGAAAGTTACTC -3’	5’- GGTGTTAGAACAAGATCATGGAT GATTTTCTTTTA -3’
CCL2 (YY1)	5’- CCCCATTTGCTCATTTGGTCTCAG -3’	5’- TGCTGGAGGCGAGAGTGCGA -3’
CXCL10 (YY1)	5’- CCTTTTCTCACACCATTCTAGATACCATC -3’	5’- CCTGCTTTGACAGGGTCAAAGATC -3’
CCL20 (YY1)	5’- CAGGGTCTTCTTCATGACAGCAC -3’	5’- ACAGCGATACAAAGGTCAGCCC -3’
TNFα (YY1)	5’- AACTTTCCAAATCCCCGCCCCC -3’	5’- CCAGCGGAAAACTTCCTTGGTG -3’
**Promoter cloning Primer**		
TNFα promoter	5’- ATGCGGTACCGAGCTCCTGGGAGATATGGC CACA -3’	5’- ATGCGATATCTCAGTGCTCATGGTGTC CTT TCCAG -3’

Table S2. Antibodies used in this study.

Antibody	Cat #	Company	Application
CXCL8	ab89336	Abcam	IHC, WB
CCL2	MABN712	Millipore	IHC, WB
CXCL10	ab8098	Abcam	IHC, WB
CCL20	ab9829	Abcam	IHC, WB
Pref-1	33697	Novus Biologicals	IHC
CD29	ab183666	Abcam	IHC
CD163	A8383	ABclonal	IHC
CD206	A8301	ABclonal	IHC
RFP	AE020	ABclonal	IHC
pIκB	2859S	Cell signaling	WB
IκB	ab7217	Abcam	WB
HA	AE008	ABclonal	WB
α/β-tubulin	2148	Cell signaling	WB
p65	ab7970	Abcam	ICC, ChIP
p50	ab7971	Abcam	ICC, ChIP
AcH3	06-599	Millipore	ChIP
p300	sc-585	Santa Cruz Biotechnology	ChIP
IL6	A11114	ABclonal	IHC
Anti-Rabbit IgG–TRITC	T6778	Sigma	IHC, ICC
Anti-Mouse IgG–FITC	F0257	Sigma	IHC
Anti-Mouse IgG–HRP	sc-2005	Santa Cruz Biotechnology	WB
Anti-Rabbit IgG–HRP	sc-2357	Santa Cruz Biotechnology	WB

IHC: Immunohistochemistry, **WB**: Western blot, **ICC**: Immunocytochemistry, **ChIP**: Chromatin Immunoprecipitation

Table S3. Chemokines and Cytokines used in this study.

Chemokine/Cytokine	Cat #	Company
CXCL8	200-08M	Pepro Tech
CCL2	300-04	Pepro Tech
CXCL10	300-12	Pepro Tech
CCL20	300-29A	Pepro Tech
IL4	200-04	Pepro Tech
IL13	200 13	Pepro Tech

Table S4. Characteristics of prostate cancer patients in tissue array used in this study.

Characteristics	N (%)
**Gender**	Male
**Mean age in year (range)**	64.98 (44-72)
**Mean preoperative PSA ng/ml (range)**	26.64 (0.5-161)
**pTNM pathologic classification**	
pT2a	1 (2.5%)
pT2b	7 (17.5%)
pT3	32 (80.0%)
**Gleason score**	
4-7	17 (42.5%)
8-10	23 (57.5%)

N: Patient number, **PSA**: Prostate-specific antigen, **pTNM**: Pathological tumor-node-metastasis

pT2a: Tumor affects one-half of one lobe or less

pT2b: Tumor affects more than one-half of one lobe but not both lobes

pT3: Tumor extends beyond the prostate

Table S5. Relationship between expression level of SFMBT2 and Gleason Score.

	SFMBT2 expression			
**Gleason Score**	**High (%)**	**Moderate (%)**	**Low (%)**	**Total cases (%)**
**= 2**	8 (88.88%)	1 (11.11%)	0 (0%)	9 (100%)
**≤ 7**	1 (5.90%)	8 (47.06%)	8 (47.06%)	17 (100%)
**≥ 8**	1 (4.34%)	2 (8.70%)	20 (86.96%)	23 (100%)

There is significant difference $P<0.001$.
